# The role of bacterial vaccines in the fight against antimicrobial resistance: an analysis of the preclinical and clinical development pipeline

**DOI:** 10.1016/S2666-5247(22)00303-2

**Published:** 2023-02

**Authors:** Isabel Frost, Hatim Sati, Pilar Garcia-Vello, Mateusz Hasso-Agopsowicz, Christian Lienhardt, Valeria Gigante, Peter Beyer

**Affiliations:** a World Health Organization, Geneva, Switzerland; b Department of Infectious Disease, Imperial College London, London, UK; c Global Antibiotic Research and Development Partnership, Geneva, Switzerland; d Unité Mixte Internationale 233 IRD-U1175 INSERM, Université de Montpellier, Institut de Recherche pour le Développement, Montpellier, France; e Epidemiology and Population Health, Department of Infectious Disease Epidemiology, London School of Hygiene & Tropical Medicine, London, UK

## Abstract

Vaccines can be highly effective tools in combating antimicrobial resistance as they reduce infections caused by antibiotic-resistant bacteria and antibiotic consumption associated with disease. This Review looks at vaccine candidates that are in development against pathogens on the 2017 WHO bacterial priority pathogen list, in addition to *Clostridioides difficile* and *Mycobacterium tuberculosis*. There were 94 active preclinical vaccine candidates and 61 active development vaccine candidates. We classified the included pathogens into the following four groups: Group A consists of pathogens for which vaccines already exist—ie, *Salmonella enterica* serotype Typhi, *Streptococcus pneumoniae, Haemophilus influenzae* type b, and *M tuberculosis*. Group B consists of pathogens with vaccines in advanced clinical development—ie, extra-intestinal pathogenic *Escherichia coli, Salmonella enterica* serotype *Paratyphi A, Neisseria gonorrhoeae*, and *C difficile*. Group C consists of pathogens with vaccines in early phases of clinical development—ie, enterotoxigenic *E coli, Klebsiella pneumoniae*, non-typhoidal *Salmonella, Shigella* spp, and *Campylobacter* spp. Finally, group D includes pathogens with either no candidates in clinical development or low development feasibility—ie, *Pseudomonas aeruginosa, Acinetobacter baumannii, Staphylococcus aureus, Helicobacter pylori, Enterococcus faecium*, and *Enterobacter* spp. Vaccines are already important tools in reducing antimicrobial resistance and future development will provide further opportunities to optimise the use of vaccines against resistance.

## Introduction

Antimicrobial resistance (AMR) is a growing threat to global health, and was associated with 4·95 million deaths in 2019 globally, more than HIV and malaria combined.^1^ AMR compromises many aspects of modern medicine beyond bacterial infectious diseases; it also affects surgery, organ transplantation, and treatment for illnesses and ailments including HIV, liver and kidney disease, cancer, and physical trauma.^2^ Vaccines can be highly effective tools in combating AMR^3^, ^4^, ^5^, ^6^, ^7^, ^8^ and have been highlighted by WHO as such.^9^, ^10^ Vaccines work through multiple mechanisms to reduce AMR.^11^ Vaccines targeting bacterial pathogens reduce infections caused by antibiotic-resistant or susceptible pathogens, and contribute to the protection of unvaccinated populations if sufficient levels of immunity are maintained.^12^ Vaccines do this by both reducing the overall burden of infectious disease, caused by resistant or susceptible bacteria, and also by reducing antibiotic use associated with bacterial or viral infections. In addition, vaccines against viral infections—respiratory infections in particular, for example vaccines against influenza—reduce inappropriate antibiotic consumption, which is one of the key drivers of AMR.^11^ In this analysis, we consider only bacterial vaccines.

Although resistance has emerged for every antibiotic that has been introduced into clinical practice, resistance to bacterial vaccines is rare and tends not to render them ineffective. However, some vaccines have been shown to select for non-vaccine serotypes, causing replacement with those serotypes not covered by the vaccine over time.^13^ For this reason pneumococcal conjugate vaccines (PCVs) with higher and higher valences have been needed to address the increase in pneumococcal disease caused by non-PCV serotype pneumococci,^14^ and antibiotic resistance can continue to emerge in these serotypes.^15^ Despite resistance emerging in PCVs, in some cases, vaccines work to increase the viable lifetime of antibiotics. For example, PCVs cover 90% of the drug-resistant strains that cause childhood disease.^16^ Prevention of antibiotic-resistant strains through the use of these vaccines allows antibiotics to continue to be effective. PCVs targeting even more strains are currently under development.

Considerable advances have been made in vaccinology over the past four decades, which have been further accelerated by the response to the COVID-19 pandemic. These advances have opened up molecular targets and made it possible to vaccinate against more pathogens. Furthermore, vaccines present an opportunity to address increasingly difficult-to-treat infections caused by AMR bacteria.

Mapping out preclinical and clinical vaccine development against pathogens of critical, high, and medium concern related to drug resistance can help to understand and articulate research and development opportunities. Additionally, mapping out development clarifies how current vaccine investments are being distributed. This Review provides a mapping of the preclinical and clinical pipeline for vaccines against the pathogens highlighted by the 2017 WHO bacterial priority pathogen list (BPPL)—in addition to *Clostridioides difficile* and *Mycobacterium tuberculosis*—combined with an analysis of the feasibility of vaccine development (appendix; Table 1, Table 2).^18^ This list of pathogens was developed by WHO to prioritise pathogens for the research and development of new antibiotics due to emerging AMR. The inclusion of *C difficile* and *M tuberculosis* follows the approach used in previous WHO pipeline reports and recognition in the BPPL report that *M tuberculosis* is an established priority for WHO.^18^

**Table 1:** Data on the preclinical and clinical development of vaccines for pathogens in three categories

		**Preclinical**	**Phase 1**	**Phase 2**	**Phase 3**
**Group A**					
*Streptococcus pneumoniae*		17	4	8	4
*Haemophilus influenzae* type b		3	1	0	3
*Salmonella enterica* serotype Typhi		8	1	1	3
*Mycobacterium tuberculosis*		20	2	7	4
**Group B**					
Extra-intestinal pathogenic *Escherichia coli*		4	1	2	1
*Clostridioides difficile*		5	1	0	1
*Salmonella enterica* serotype Paratyphi		4	1	1	1
*Neisseria gonorrhoeae*		2	0	0	1
**Group C**					
Enterotoxigenic *E coli*		10	4	2	0
*Shigella* spp		10	6	2	0
	*Shigella flexneri*	..	3	1	0
	*Shigella sonnei*	..	0	1	0
	*S flexneri* and *S sonnei*	..	3	0	0
Non-typhoidal *Salmonella*		5	1	0	0
*Klebsiella pneumoniae*		5	1	0	0
*Campylobacter* spp		4	0	0	0

Group A are pathogens with vaccines that are already licensed. Group B are pathogens with vaccines in late-stage clinical trials with high development feasibility. Group C are pathogens with vaccine candidates either in early clinical trials or with moderate to high feasibility of vaccine development.

**Table 2:** Definition of feasibility

	**Definition**
Biological feasibility	Considers progression of clinical development; existence of immunity from natural exposure; current understanding of mechanisms of immunity; and the likelihood of a vaccine protecting against most pathogenic strains
Product development feasibility	Considers the existence of established animal and in-vitro models to facilitate vaccine development; the ease of clinical development and setting a late-stage clinical trial; and the availability of human challenge models if these are likely to be required
Access and implementation feasibility	Considers the possibility of implementation within existing delivery systems, in particular childhood immunisation programmes; commercial attractiveness and whether there are likely to be high-income markets to support tiered pricing; the clarity of the licensure and policy decision pathway; and the ease of uptake and acceptability in target populations

Each aspect was rated from very low to very high feasibility.^17^

## Results and discussion: reviewing the pipeline

**Figure 1:**
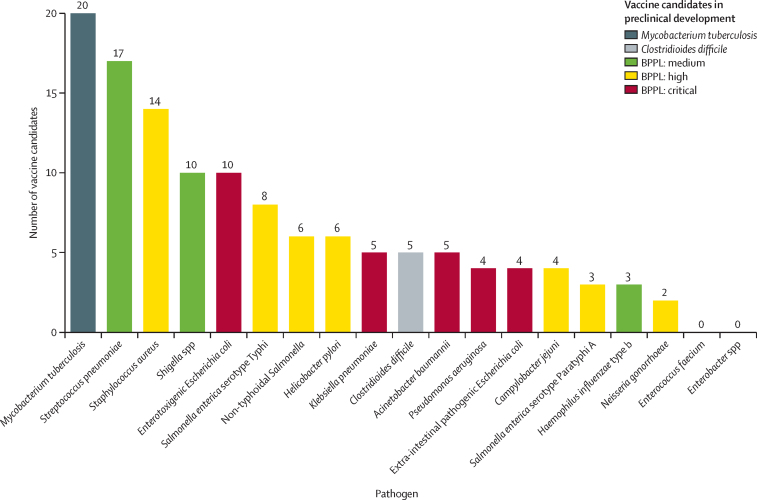
Vaccine candidates in preclinical development, categorised by pathogen and type BPPL=bacterial priority pathogen list.

**Figure 2:**
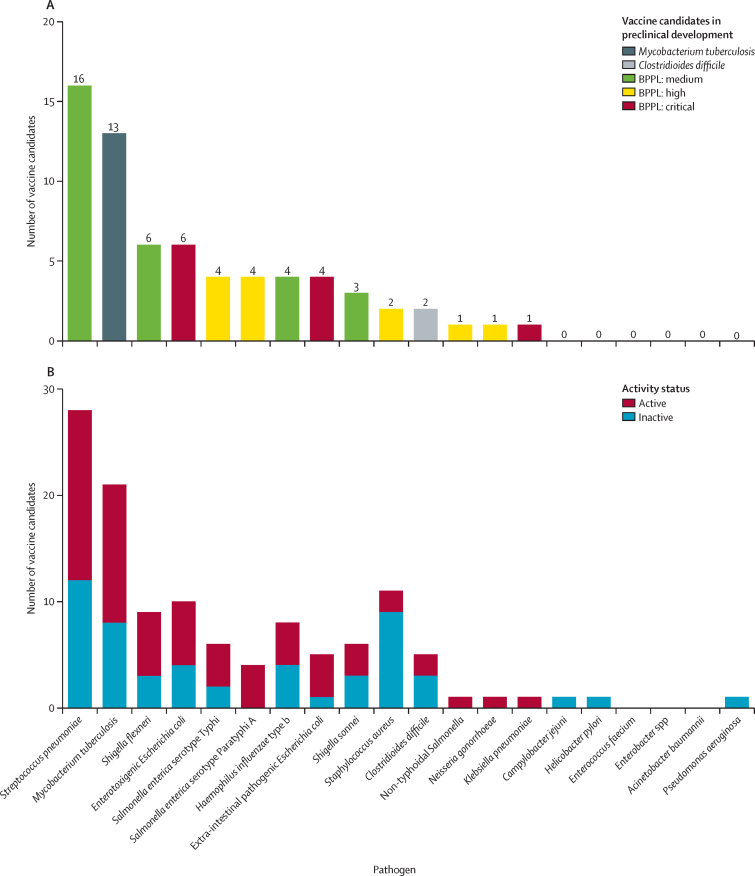
Vaccine candidates in active clinical development (A) Total number of vaccine candidates in active clinical development by pathogen. (B) Total number of candidates both in active clinical development and that have become inactive or discontinued over the past 10 years, by pathogen. Note that some vaccine candidates are double counted here as they target more than one pathogen. Pathogen type refers to status as defined by the WHO BPPL.^14^ BPPL=bacterial priority pathogen list.

A total of 94 vaccine candidates were identified in active preclinical development (figure 1) and 61 vaccine candidates were identified in active clinical development (figure 2). Most of the vaccines that are in preclinical development target *M tuberculosis* (n=20), *Streptococcus pneumoniae* (n=17), and *Staphylococcus aureus* (n=14). For vaccines in the clinical development stage, most candidates target *S pneumoniae* (n=16), *M tuberculosis* (n=13), *Shigella flexneri* (n=6), and enterotoxigenic *Escherichia coli* (n=6). There are currently no vaccines under active clinical development that target *Campylobacter jejuni, Helicobacter pylori, Enterococcus faecium, Enterobacter* spp, *Acinetobacter baumannii*, or *Pseudomonas aeruginosa*. However, apart from *E faecium* and *Enterobacter* spp, all of these pathogens have vaccines in preclinical development.

## Group A: pathogens with vaccines that are already licensed

Vaccines targeting *S pneumoniae, Haemophilus influenzae* type b, and *Salmonella enterica* serotype Typhi (*S* Typhi) are already licensed. Research to improve these vaccines continues and vaccine candidates in clinical development against *S pneumoniae* outnumber any of the other pathogens considered. Current BCG vaccines, which are licensed against *M tuberculosis*, continue to save the lives of many children each year. However, BCG vaccines do not provide adequate protection against pulmonary tuberculosis, a disease associated with high burden in many parts of the world.

### S pneumoniae

A 23-valent pneumococcal polysaccharide vaccine has been licensed for use in children (aged ≥2 years) and adults since 1983,^19^ and many more vaccines have come to the market since. Two PCVs have been on the market since 2009; these are 10-valent and 13-valent vaccines recommended by WHO in childhood immunisation schedules for use in children younger than 5 years.^20^ These vaccines have reduced the incidence of infections caused by drug-susceptible and drug-resistant *S pneumoniae*. Observational studies show that in just 5 years the first PCV introduced in the USA reduced invasive pneumococcal disease caused by multidrug-resistant *S pneumoniae* by 84% in children younger than 2 years. Penicillin-resistant invasive pneumococcal disease was also reduced by 49% in adults older than 65 years, probably due to reduced transmission from children, as this group did not receive the vaccine.^12^, ^17^ PCV10 and PCV13 are both widely used and cover serotypes 10 and 13, respectively. Although more than 100 *S pneumoniae* serotypes exist, most disease-causing serotypes are covered by current vaccines. However, as vaccines reduce the incidence of vaccine serotypes, replacement serotypes, which are not covered by current vaccines, have increased in some populations.^14^ Under current coverage, PCVs prevent an estimated 23·8 million cases of antibiotic-treated illness among children younger than 5 years in low-income and middle-income countries (LMICs) every year.^21^

*S pneumoniae* was identified as one of the leading causes of death related to AMR infections in 2019, and it was the number one cause of death due to resistant infections in sub-Saharan Africa.^1^ Despite the availability of effective vaccines against *S pneumoniae*, worldwide coverage remains low at approximately 40% in children younger than 5 years.^22^ Uptake needs to be supported by global policies and interventions to improve access and affordability, particularly in low-resource settings where the burden of infectious diseases is highest. Research goals include reducing the manufacturing costs for PCVs, increasing serotype coverage and tailoring to local epidemiology, and developing new methods for protein conjugation and vaccine manufacturing.^3^

### *H influenzae* type b

Effective vaccines against *H influenzae* type b have been available since the 1990s and WHO recommends the inclusion of *H influenzae* type b vaccines in infant immunisation programmes.^23^ Through their use in children younger than 5 years, invasive disease caused by *H influenzae* type b is close to being eliminated in high-income countries.^3^ *H influenzae* type b vaccines have also contributed to reducing the prevalence of some drug-resistant strains.^24^ Increasing the uptake of *H influenzae* type b vaccines globally would reduce infections caused by drug-resistant *H influenzae* type b.

### *S* Typhi

More than 20 vaccines have been authorised against *S* Typhi.^3^ The WHO Strategic Advisory Group of Experts on Immunization recommends the existing WHO-approved typhoid conjugate vaccine (TCV) in favour of unconjugated Vi polysaccharide vaccine and live attenuated Ty21a vaccine due to TCV's improved immune response, longer expected duration of protection, and its suitability for use in those younger than 2 years.^25^ TCV has been used successfully in Pakistan to combat outbreaks of typhoid caused by drug-resistant *S* Typhi.^26^ Current research aims to incorporate multiple pathogen targets alongside *S* Typhi, which would make the value proposition of the vaccine even more favourable, and seeks to better understand the effect of vaccination on long-term pathogen carriage. Models suggest that the introduction of routine immunisation with TCV at age 9 months, with a catch-up campaign to age 15 years, would avert 21·2 million cases and 342 000 deaths from multidrug-resistant typhoid over the 10 years after introduction in the 73 countries eligible for Gavi, the Vaccine Alliance support.^27^

### M tuberculosis

The BCG vaccine, active against *M tuberculosis*, is the most widely administered vaccine globally.^28^, ^29^ WHO continues to recommend BCG vaccination in countries and settings where the tuberculosis burden is high.^30^ It was developed 100 years ago and continues to effectively avert thousands of paediatric deaths every year. BCG provides long-lasting strong protection against miliary and meningeal tuberculosis, the most deadly forms of tuberculosis in children, but it is less effective for the prevention of pulmonary tuberculosis in adults. Importantly, BCG does not prevent reactivation of latent pulmonary infection—the principal source of bacillary spread in the community—and, therefore, has no activity against tuberculosis transmission.^31^ Given the high global burden of tuberculosis, and concerning levels of drug resistance, a new more effective tuberculosis vaccine is greatly needed. Model estimates suggest a novel vaccine could avert 115 000 deaths due to rifampicin-resistant tuberculosis between 2020 and 2035.^32^

At present, there are four vaccine candidates in phase 3 clinical trials targeting *M tuberculosis*. VPM1002 is a prophylactic recombinant BCG vaccine, currently in phase 3 trial in newborn infants to assess its efficacy, safety, and immunogenicity against *M tuberculosis* infection. The trial is scheduled to be completed in June, 2025 (NCT04351685). A phase 2/3 trial of VPM1002 for preventing tuberculosis recurrence in treated patients is also underway in India. GamTBvac is a prophylactic recombinant vaccine under development by the Russian Ministry of Health, that was scheduled to begin a phase 3 trial in November, 2021 (NCT04975737). MTBVAC is a live attenuated *M tuberculosis* candidate for which a phase 3 trial is planned to start in July, 2022, among newborn babies in sub-Saharan Africa (NCT04975178). Lastly, Immuvac is a therapeutic vaccine that uses a heat-killed *Mycobacterium indicus pranii* and is in a phase 3 trial in India.

There are a total of seven vaccine candidates in phase 2 clinical trials against tuberculosis, three in phase 2a and four in phase 2b. Among these, M72/AS01E is a subunit candidate, composed of an immunogenic fusion protein (M72) derived from two *M tuberculosis* antigens (MTB32A and MTB39A), and the adjuvant AS01E.^33^ A phase 2b clinical trial reported that M72/AS01E had an efficacy of nearly 54·0% in preventing active tuberculosis over 3 years in adults infected with *M tuberculosis.*^34^ A multicentre phase 3 trial is currently in preparation. H56:IC31 is a preventive recombinant subunit vaccine consisting of three antigens (ie, Ag85B, ESAT-6, and Rv2660c) currently studied in South Africa and Tanzania for prevention of recurrence (NCT03512249). DAR-901 takes a heat inactivated whole-cell approach and is prophylactic.^33^, ^34^ RUTI is a vaccine composed of *M tuberculosis* cell fragments and is a therapeutic vaccine candidate.

## Group B: pathogens with vaccines in late-stage clinical trials with high development feasibility

**Figure 3:**
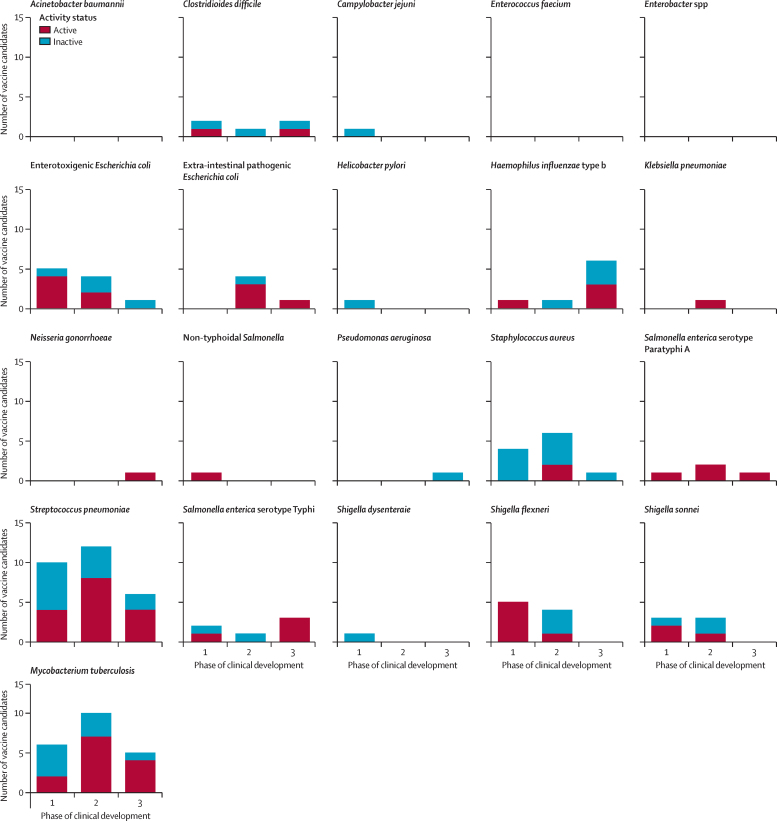
Number of vaccine candidates by phase of clinical development and continued activity

Vaccine candidates are shown by phase of clinical development in figure 3. There are currently vaccine candidates in phase 3 of development targeting: extra-intestinal pathogenic *E coli, Salmonella enterica* serotype Paratyphi A (*S* Paratyphi A), *Neisseria gonorrhoeae,* and *C difficile.* Candidates in phase 3 are the most clinically advanced, and therefore most likely to be available in the near future.

### Extra-intestinal pathogenic *E coli*

*E coli* is the leading cause of deaths associated with antibiotic-resistant bacterial pathogens.^1^ Infections caused by *E coli* have multiple manifestations, including both extra-intestinal pathogenic *E coli* and enterotoxigenic *E coli*. Extra-intestinal pathogenic *E coli* are a common cause of highly resistant hospital-acquired infections. There are multiple challenges associated with developing vaccines against hospital-acquired infections, including the relatively low incidence of infections caused by extra-intestinal pathogenic *E coli* in vaccine target populations (panel 1). An extra-intestinal pathogenic *E coli* vaccine might have different target populations depending on whether it targets invasive sepsis in infants or older adults, or urinary tract infections, in those with recurrent, complicated, or catheter-associated urinary tract infections. As *E coli* is a commensal pathogen, the effect on the broader microbiota and the potential consequence of disrupting it would also need to be assessed. There are four vaccine candidates in clinical development targeting extra-intestinal pathogenic *E coli*. The most advanced in development is ExPEC9V, a nine-valent-O-polysaccharide conjugate vaccine, currently in a phase 3 clinical trial (NCT04899336) against invasive disease, which is expected to be completed in May, 2027.

Panel 1 **Challenges of developing vaccines against pathogens causing hospital-acquired infections**

Many of the WHO bacterial priority pathogens cause hospital-acquired infections, in particular *Acinetobacter baumannii, Klebsiella pneumoniae, Pseudomonas aeruginosa, Staphylococcus aureus*, extra-intestinal pathogenic *Escherichia coli, Clostridioides difficile, Enterococcus faecium*, and *Enterobacter* spp. There are multiple challenges^35^ for the clinical development of vaccines against these pathogens:

• The relatively low incidence of infections in the vaccine target population makes efficacy trials prohibitively large in terms of trial sites and patient numbers, and expensive^3^
• Potential target populations tend to be critically ill, with multiple comorbidities and severely compromised immune systems, making clinical endpoints hard to establish^36^, ^37^
• Identification of populations at risk of hospital-acquired infections at intensive care unit admission is impracticable, due to the little time available to mount an effective immune response^38^
• At present there is no regulatory or policy precedent for a vaccine against hospital-acquired infections

Despite these limitations, it could be possible to identify patients at high risk, such as those scheduled for elective surgery or hospital treatment, which would allow enough time for their immune systems to respond to vaccine administration. Given these challenges, regulators could explore the use of correlates of protection in phase 3 trials followed by the collection of post-licensure effectiveness data and real-world evidence. However, correlates of protection are currently absent for these pathogens. Combination vaccines might require fewer participants in clinical trials than single-target vaccines as multiple causes of disease are being targeted and incidence will be higher, which could help facilitate trials.

### C difficile

Infections caused by *C difficile* are often difficult to manage with antibiotics, and alternative strategies are greatly needed. Clinical trial designs must take into account that the clinical endpoint is diarrhoea, which has multiple causes, particularly in older patients (aged >65 years), who are the vaccines' main target population (panel 1). Data from vaccine candidates that are currently in clinical development suggest that these agents could reduce symptomatic disease; however, *C difficile* could continue to be shed by the host.^39^ Further challenges to vaccine development against *C difficile* include the recruitment of patients in appropriate target populations. Many of these patients are older, severely ill, or have other comorbidities, which creates difficulties in establishing and evaluating clinical endpoints. There are currently three vaccine candidates against *C difficile* in clinical trials. PF-06425090 is a recombinant toxin vaccine targeting *C difficile*, consisting of genetically and chemically detoxified TcdA and TcdB toxins.^40^, ^41^ PF-06425090 did not meet its primary endpoint of preventing *C difficile* infections in a phase 3 trial (NCT03090191); however, disease severity was reduced.^42^ The study recruited 17 500 participants and the final analysis was done after accruing 42 cases of *C difficile* infection within 4 years of primary vaccination, reflecting the economic and logistical challenges of conducting studies on hospital-acquired infections.

### *S* Paratyphi

The global mortality burden of drug-resistant *S* Paratyphi was estimated to be 20 224 associated deaths in 2019, with 4106 deaths directly attributed to it.^1^ However, the relatively low incidence in vaccine target populations would require large and long-lasting clinical trials or a controlled human infection model. A controlled human infection model has been developed but has not yet been used clinically for vaccine evaluation.^43^ A vaccine would most likely be co-administered in combination with TCV, improving the value proposition of both vaccines. Despite differences between *S* Typhi and *S* Paratyphi A, in particular the absence of Vi capsular polysaccharide in *S* Paratyphi A, the successful development of TCV suggests a vaccine against *S* Paratyphi A is also possible. Three vaccines are in clinical development against *S* Paratyphi. A phase 3 trial of the O:2, 12-TT conjugate vaccine is currently being conducted in China.

### N gonorrhoeae

There are multiple challenges to vaccine development against *N gonorrhoeae*, including the absence of known correlates of protection, absence of immunity from natural exposure, poor understanding of immunity, and the existence of multiple pathogenic strains. Despite these challenges conserved antigenic targets have been identified. There is evidence to suggest the Bexsero vaccine (GlaxoSmithKline, Kundl, Austria), developed and licensed to prevent group B meningococcal infections, provides some protection against gonorrhoea. MenB given to children and young adults was associated with an approximately 30% reduction in gonorrhoea diagnoses.^44^ Two large clinical trials are underway to investigate the efficacy of Bexsero against *N gonorrhoeae*; however, phase 3 trial results have been delayed by the COVID-19 pandemic (NCT04415424).^45^ Modelling estimates suggest that gonococcal prevalence could be reduced by 40% by a vaccine with only 20% efficacy if it were given to all children aged 13 years.^46^ Another model in men who have sex with men (MSM) suggested that a vaccine would need a minimum efficacy of 90% and a fixed uptake of 40% to completely prevent AMR development.^44^ A third model in MSM estimated that even in a worst-case scenario, in which untreatable gonorrhoea infections emerge, the WHO 2016–30 target of a 90% reduction in gonorrhoea incidence would be achievable, under the condition that all MSM attending sexual health clinics receive a vaccine that offers at least 52% protection for more than 6 years.^47^

## Group C: pathogens with vaccine candidates either in early clinical trials or with moderate to high feasibility of vaccine development

The pathogens in Group C are associated with moderate feasibility of vaccine development and include Enterotoxigenic *E coli, Klebsiella pneumoniae*, non-typhoidal *Salmonella, Campylobacter* spp, and *Shigella* spp. However, given the early stages of development of vaccines against these targets, no vaccine is likely to be available on the market soon.

### Enterotoxigenic E coli

There are vaccine candidates against enterotoxigenic *E coli* and *Shigella* spp that are in phase 2 of clinical development. Mortality estimates are established for enterotoxigenic *E coli, Campylobacter* spp, and *Shigella* spp; however, long-term morbidity data on stunting and cognitive impairment are scarce. Better data on the economic effects of these infections are also needed to assess vaccine value and potential impact. Much of the disease burden is in LMICs, which reduces commercial attractiveness for the private sector. Three of the six vaccines in clinical trials against enterotoxigenic *E coli* also target *Shigella* spp and several candidates in preclinical development target even more combinations of pathogens. Such a multipronged approach would improve the value proposition of a vaccine. Evidence that the Dukoral cholera vaccine (Valneva, Solna, Sweden) provides 3 months' protection against some enterotoxigenic *E coli* strains suggests that an enterotoxigenic *E coli* vaccine is also possible.^48^, ^49^ Despite the diversity of enterotoxigenic *E coli* strains, up to 80% of those that cause disease could be covered by a vaccine targeting heat-labile toxoid and colonisation factor antigens.^50^ Two vaccines in phase 2 are being developed against enterotoxigenic *E coli*: ETVAX/dmLT, an inactivated whole cell vaccine (PACTR202010819218562);^51^ and CfaE+mLT, a recombinant subunit vaccine (NCT01922856).^52^

### Shigella *spp*

A vaccine against *Shigella* spp might be used in multiple target populations, including infants (≤24 months) living in endemic regions, travellers to endemic regions, MSM, and military personnel. Although multiple strains of disease-causing *Shigella* spp exist, research suggests that more than 80% of these would be covered by a four-valent conjugate vaccine. More research is needed to ascertain whether conjugate vaccines will gain a sufficient immune response in the target population of those younger than 3 years; however, adjuvants could improve this response. Two vaccines targeting *Shigella* spp have reached phase 2 development. GlycoShig3 is a glycoconjugate subunit vaccine against *S flexneri* (NCT04602975; estimated study completion is Sept 30, 2023).^53^ WRSS2/WRSS3 is a live attenuated whole-cell vaccine targeting *Shigella sonnei* (NCT04242264; estimated study completion is 2023).^54^ There are also several vaccines in early stages of development that combine *Shigella* spp and enterotoxigenic *E coli* as targets.

### Non-typhoidal *Salmonella*

The global burden of non-typhoidal *Salmonella* associated with drug-resistant infection was estimated to be 27 133 deaths in 2019.^1^ The development of a vaccine against non-typhoidal *Salmonella*, appears biologically feasible and would help to reduce the substantial burden and mortality of invasive non-typhoidal *Salmonella* disease.^3^ A large proportion of this burden occurs in Africa and is caused by the *S* Typhi and *Salmonella enterica* serotype Enteritidis.^55^, ^56^ Coverage of these serotypes could prevent most disease-causing strains. iCVD1000 is a trivalent conjugate vaccine that targets *S* Typhi, and non-typhoidal serotypes and Enteritidis. A phase 1 trial is scheduled for completion in September, 2022 (NCT03981952).

### K pneumoniae

*K pneumoniae* has been identified by WHO as a critical priority resistance threat^18^ and one of six pathogens with the highest mortality due to antibiotic-resistant infections.^1^ Multiple challenges are associated with the development of vaccines against highly resistant hospital-acquired infections (panel 1). *K pneumoniae* is associated with a high burden of neonatal sepsis in low-resource settings and a maternal vaccine has been suggested to combat this burden. Commercial attractiveness is likely to be low as much of the burden is in LMICs; however, if there is also a market in high-income countries tiered pricing based on country income levels could make development more economically feasible. A further hurdle is the design and implementation of clinical trials involving neonates, which can be challenging to conduct. A tetravalent bioconjugated vaccine candidate, KlebV4, is being assessed with and without the AS03 adjuvant in a phase 1/2 trial (NCT04959344).

### Campylobacter *spp*

*Campylobacter* spp are the leading cause of bacterial gastroenteritis in high-income countries.^57^ There are currently no vaccines in clinical development against *C jejuni*. Some vaccine candidates have been developed to the clinical stage in the past, but they have all been unsuccessful. Such a vaccine would have markets in high-income and low-income countries and potential target populations in infants and international travellers. There is a conjugate vaccine that has been developed for use in cows, which has shown *Campylobacter* spp to be a viable target for vaccines.^58^

## Group D: pathogens with a small number of or no vaccine candidates in the pipeline or low vaccine development feasibility in the near future

Pathogens in group D are associated with low feasibility of vaccine development and include the priority pathogens *S aureus*, with one vaccine candidate in phase 2; *A baumannii, P aeruginosa*, and *H pylori,* which have no vaccine candidates in clinical development; and *E faecium* and *Enterobacter* spp, which do not have any vaccine candidates in preclinical or clinical development. There are vaccine candidates in preclinical development for all pathogens in the BPPL except for *Enterobacter* spp and *E faecium*. This exemption reflects the restricted feasibility of developing a vaccine against these two pathogens.^59^ Many of the pathogens of critical priority in the BPPL commonly cause difficult-to-treat hospital infections. An overview of challenges of developing vaccines against pathogens causing hospital-acquired infections is shown in panel 1.

One vaccine candidate is in phase 2 clinical trials against *S aureus*; it is a recombinant toxic shock syndrome toxin-1 variant vaccine. The vaccine candidate was reported to be safe and immunogenic in a phase 1 trial^60^ and a phase 2 trial was completed in January, 2021, the results of which are yet to be published (NCT02814708). Despite relatively high investment from industry, many of the candidates that target *S aureus* that have been developed, have later been unsuccessful, including nine candidates over the last 10 years that are included in this study. A four-antigen vaccine was terminated during phase 2b in 2019 due to futility (NCT02388165).^4^ The V710 IsdB vaccine candidate proved unsuccessful after the termination of its phase 3 trial. This cancellation came after the interim analysis reported increased mortality and adverse side-effects among participants who had taken the vaccine candidate and later developed an *S aureus* infection (NCT00518687). CP5-Epa and CP8-Epa vaccines also reached phase 3 of clinical trials, but are no longer under development.^61^

Although there are three candidates in clinical development against *S aureus*, this pathogen has been categorised in a low feasibility category due to extensive challenges in developing a vaccine against the pathogen in the past. There are multiple challenges to developing a successful vaccine against *S aureus* (panel 1). First, it is unclear which antigen targets would enable a vaccine to provide protection against *S aureus* infection.^4^ Correlates of protection are scarce, and vaccines that have shown protection in rodent models have subsequently been unsuccessful in clinical trials. Although multiple candidate monoclonal antibodies targeted against *S aureus* have been tested, these have also proven ineffective in clinical trials.^62^ Second, intermittent colonisation with *S aureus* occurs in approximately two-thirds of the population; however, exposure does not appear to confer natural immunity.^63^, ^64^ Finally, the diseases caused by *S aureus* are diverse, including bacteraemia, skin infections, pneumonia, and others, and it is not clear whether a single vaccine would be protective against multiple clinical syndromes.^65^

## Development approaches and future trends in research and development

**Figure 4:**
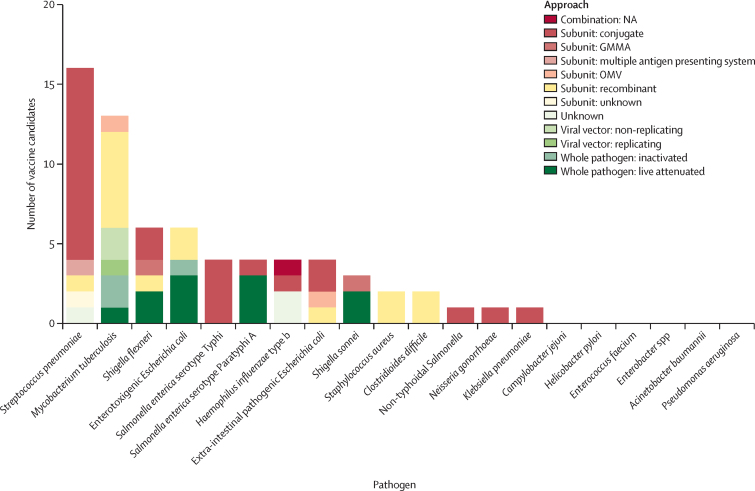
Number of vaccine candidates in active clinical development by approach NA=not applicable. GMMA=generalised modules for membrane antigens. OMV=outer membrane vesicles.

The vaccine candidates in clinical development use many different mechanistic approaches (figure 4). The biological approach adopted by clinical candidates was not recorded as, in many cases, this information was unavailable. However, approaches in preclinical development are likely to be even more diverse. Vaccine development during the COVID-19 pandemic carries lessons for vaccines to tackle AMR. Global recognition of COVID-19 as an urgent threat and the lifesaving potential of vaccines converged to global prioritisation, investment, and research, resulting in a reduced timeline for licensure and use. This acceleration was facilitated by an increase in available resources, and optimal approaches to both regulating and doing clinical trials in parallel. A further consequence of the COVID-19 pandemic has been advancements in vaccine technology, in particular the development of mRNA vaccines. Although only one clinical mRNA candidate targeting *M tuberculosis* was identified in this Review, many more candidates are likely to emerge, potentially targeting AMR pathogens. The advantages of mRNA vaccine technology include that they are less costly to manufacture and can be developed and scaled up for production more rapidly than other vaccine approaches.^66^, ^67^ The expression of the antigen occurs in the body of the vaccine recipient, avoiding complex, costly, and time-consuming steps in manufacture compared with other vaccines.^68^ However, research challenges to the development of mRNA vaccines against AMR pathogens include thermostability and the need for ultra-cold chain and storage. At present, multiple antigens have not been combined into a single vaccine and all currently licensed mRNA vaccines target viral pathogens.^68^ It is also important to highlight that mRNA technology is not going to solve other scientific challenges in vaccine development—eg, when it is unclear how the human immune response against a specific pathogen is triggered.

Another research area that would improve the value proposition of vaccines against AMR pathogens is the development of combination vaccines with multiple antigen targets. In some disease areas combination vaccines are already being developed, for example *Shigella*–enterotoxigenic *E coli* vaccine candidates and candidates targeting multiple serotypes of *Salmonella*. A vaccine targeting multiple key pathogens, which cause hospital-acquired infections, would allow for easier efficacy trials due to relatively increased incidence of disease in the patient population. Vaccines targeting multiple pathogens is already an area being explored for some preclinical candidates, for example the Paragon Novel Vaccine, which targets *S aureus*, enterotoxigenic *E coli*, and extra-intestinal pathogenic *E coli*. Another example is KapaVax, an inactivated whole-cell vaccine against *K pneumoniae, P aeruginosa*, and *A baumannii.*

## Recommendations

**Figure 5:**
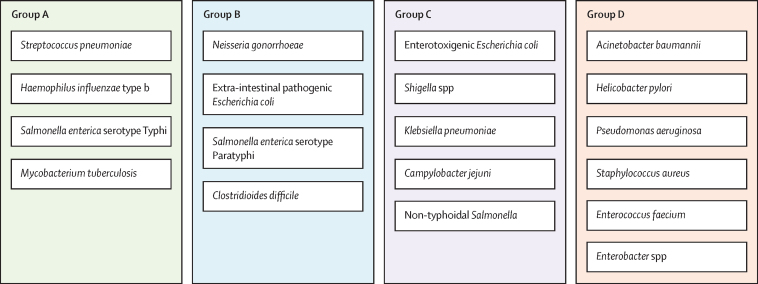
Categorisation of priority pathogens as targets for vaccination Group A contains pathogens with vaccines that are already licensed. Group B contains pathogens with vaccines in late-stage clinical trials with high development feasibility. Group C contains pathogens with vaccine candidates either in early clinical trials or with moderate to high feasibility of vaccine development. Group D contains pathogens with a small number or no vaccine candidates in the pipeline and low vaccine development feasibility in the near future.

This analysis has classified the pathogens on the BPPL, *M tuberculosis,* and *C difficile* for AMR into four distinct groups by feasibility (figure 5). Groupings were based on matching the progression of vaccine candidates in clinical and preclinical development and assessments of the feasibility of generating a vaccine. Feasibility assessments were based on analyses of biological feasibility, product development feasibility, and access and implementation feasibility. These groups and our recommendations for each are outlined in panel 2.

Panel 2 **Recommendations and summary results of vaccine feasibility for pathogens on the WHO bacterial priority pathogen list for antimicrobial resistance in addition to Mycobacterium tuberculosis and Clostridioides difficile**

**Group A**

*Pipeline feasibility (very high)*

Constitutes antimicrobial resistance (AMR) priority pathogens for which licensed vaccines already exist. Group A pathogens are associated with a very high feasibility of vaccine development and include *Salmonella enterica* serotype Typhi, *Streptococcus pneumoniae, Haemophilus influenzae* type b, and *M tuberculosis*.

*Recommendation*

Increase the coverage of authorised vaccines in line with WHO immunisation targets to maximise impact on AMR. Accelerate the development of more effective vaccines against tuberculosis.

**Group B**

*Pipeline feasibility (high)*

Constitutes AMR priority pathogens for which a vaccine candidate is in late-stage development (phase 3) and vaccines would be suitable to target AMR infections caused by these priority pathogens in the coming years. Group B pathogens are associated with a high feasibility of vaccine development and include extra-intestinal pathogenic *Escherichia coli, Salmonella enterica* serotype Paratyphi A, *Neisseria gonorrhoeae*, and *C difficile*.

*Recommendation*

Accelerate the development of vaccines for these pathogens.

**Group C**

*Pipeline feasibility (moderate)*

Constitutes AMR priority pathogens for which a vaccine candidate has either been identified in early clinical trials or been identified as a feasible vaccine target during expert review. For these pathogens, vaccines might be feasible solutions to target AMR infections. These pathogens are associated with moderate feasibility of vaccine development and include enterotoxigenic *E coli, Klebsiella pneumoniae*, non-typhoidal *Salmonella, Campylobacter* spp, and *Shigella* spp. Given the early stages of development, no vaccine for these pathogens will be available on the market soon.

*Recommendation*

Continue the development of a vaccine for these pathogens and expand knowledge of potential for vaccine use and impact and other tools to combat the AMR threat.

**Group D**

*Pipeline feasibility (low)*

Constitutes AMR priority pathogens for which no vaccine candidate has been identified in clinical development; therefore, vaccines are not a feasible solution to target AMR infections in the foreseeable future. These pathogens are associated with low feasibility of vaccine development and include the priority pathogens *Acinetobacter baumannii, Pseudomonas aeruginosa, Enterobacter* spp, *Enterococcus faecium, Staphylococcus aureus*, and *Helicobacter pylori*. Research and investment should explore alternative methods of control, including treatments and effective infection prevention, and should ensure access to clean water, adequate sanitation, and hygiene facilities. This focus is even more urgent as the drug development pipeline for *A baumannii* and *P aeruginosa* is also currently insufficient to adequately address the burden posed by these critical pathogens.

*Recommendation*

Focus on other prevention and control tools to combat the AMR threat linked to these priority pathogens. Conduct basic research to understand biological and product development barriers and ultimately facilitate vaccine development.

## Conclusion

This report provides a mapping of vaccine candidates, against pathogens prioritised due to AMR. This analysis of the preclinical pipeline is a non-exhaustive list of preclinical research at the time of writing this Review. Due to the high uncertainty and turnover of programmes in this phase of drug development, the preclinical dataset is highly dynamic. In general, failure rates in vaccine development are high. The average vaccine requires a development timeline of 11 years from the preclinical phase and has a market entry probability of 6%.^69^ Thus, many of the vaccine candidates described in this Review are likely to be unsuccessful in clinical development. For those vaccines that make it to the market, public health driven vaccination strategies will have to be developed for the appropriate target populations. This step often proves challenging for some of the pathogens, particularly those targeting hospital-acquired infections (panel 1). Finally, if vaccines become available, they will only mitigate AMR if they are available to those who would benefit from them. Three pathogens on the BPPL already have licensed, highly effective vaccines: *S pneumoniae, S* Typhi, and H influenzae type b. Despite the existence of an effective vaccine, 122 000 deaths were attributed to resistant *S pneumoniae* globally in 2019 and, of these, 40 400 occurred in sub-Saharan Africa.^1^ It is essential that these tools to combat AMR, and save lives, are made available globally once developed. Access to PCV vaccines continues to be low, and the vaccine is costly for many countries, resulting in global coverage that has reached only 40% of the target population.^22^ Funding for vaccination campaigns, and investment for research into cheaper, more cost-effective methods of manufacture and distribution, and novel methods of delivery and administration are needed to ensure vaccines reach those who need them, in the most efficient way.

For some pathogens, the absence of dual markets across high-income and low-income countries is challenging for financing vaccine development (eg, for *Shigella* spp); however, the development and WHO recommendation of a vaccine against malaria in October, 2021, has shown that financing without a dual market is possible. Some ways in which financing without a dual market can be facilitated include mechanisms for pooled procurement, such as Gavi financing and country ownership in co-financing; risk-sharing mechanisms; and end-to-end planning of policy, regulatory, and financing pathways. Access to vaccines is not solely an issue of equity; populations living in areas with high levels of infectious disease provide opportunities for resistant pathogens to emerge and propagate, irrespective of country borders.^70^ These clinically vulnerable populations inevitably need to consume antibiotics to treat infections that might have been prevented through vaccination or access to clean water, appropriate sanitation, and hygiene facilities, further exacerbating a cycle of poverty, infectious disease, and AMR.

## Search strategy and selection criteria

The method that was followed was taken from previous WHO analyses of the antimicrobial pipeline. A search of clinical trials was performed in both ClinicalTrials.gov and the International Clinical Trials Registry Platform. Search terms consisted of the pathogen name and the word “vaccine”. In addition, national databases in Russia and Japan were also searched by local experts. A review of peer-reviewed and grey literature was also done to look for candidates. PubMed was searched from 2010 to 2021, for papers in English, using the following search terms for each pathogen: ((vaccine) AND ((candidate) OR (pipeline) OR (research) OR (landscape))) AND (pathogen name) AND ((phase 1) OR (phase 2) OR (phase 3) OR (clinical)). Titles and abstracts were then screened for relevance before papers were analysed. Consulted grey literature included reports, documents and slides from WHO Product Development for Vaccines Advisory Committee meetings, key reports, the WHO tracker database (discontinued at the end of 2018), and any other grey literature that was referenced or suggested by experts. Data were also included from the WHO Global Observatory on Health Research and Development, provided by AdisInsight, and the Dynamic Dashboard from the Global Antimicrobial Resistance Research and Development Hub. Data including the vaccine candidate name, clinical trials, route of administration, approach, condition targeted, and sponsor were extracted and cleaned to remove duplicates.

## Declaration of interests

We declare no competing interests.
