# Supplementary material for: The role of bacterial vaccines in the fight against antimicrobial resistance: an analysis of the preclinical and clinical development pipeline
Source: Lancet Microbe. 2023 Feb;4(2):e113–25. doi: 10.1016/S2666-5247(22)00303-2 (PMC9892012; doi:10.1016/S2666-5247(22)00303-2)
Supplement: Supplementary appendix [file mmc1.pdf]

THE LANCET
Microbe

Supplementary appendix

This appendix formed part of the original submission. We post it as supplied by the authors.

Supplement to: Frost I, Sati H, Garcia-Vello P, et al. The role of bacterial vaccines in the fight against antimicrobial resistance: an analysis of the preclinical and clinical development pipeline. *Lancet Microbe* 2022; published online Dec 14. https://doi.org/10.1016/S2666-5247(22)00303-2.

Appendix

Clinical data

Pathogen	AllPathogens	PathogenType	Candidate Vaccine	Approach1	Approach2	Prophylactic Therapeutic	Administration Route	Lead Developer	Developer Type	Lead Developer Country	Phase	Activity Status
C. jejuni	Campylobacter jejuni	PPL: High	Capsule conjugate campylobacter vaccine (CJCV)	Subunit	Conjugate	Prophylactic	Parenteral	Naval Medical Research Center; Walter Reed Army Institute of Research (WRAIR)	Government	United States	1	Inactive
C. difficile	Clostridiodes Difficile	C. difficile	CDVAX	Subunit	Recombinant	Prophylactic	Oral	Royal Holloway University	Academia	United Kingdom	1	Discontin ued
C. difficile	Clostridiodes Difficile	C. difficile	GSK2904545A +/- adjuvant AS01B (CDIFF Ag - GlaxoSmithKline)	Subunit	Recombinant	Prophylactic	Parenteral	GlaxoSmithKline, plc	Private Sector	United Kingdom	1	Active
C. difficile	Clostridiodes Difficile	C. difficile	PF-06425090 (+/- adjuvant)	Subunit	Recombinant	Prophylactic	Parenteral	Pfizer	Private Sector	United States	3	Active
C. difficile	Clostridiodes Difficile	C. difficile	VLA84 (IC84)	Subunit	Recombinant	Prophylactic	Parenteral	Valneva Austria GmbH	Private Sector	Austria	2	Inactive
C. difficile	Clostridiodes Difficile	C. difficile	Cdiffense (ACAM-CDIFF)	Subunit	Toxoid	Therapeutic	Parenteral	Sanofi Pasteur	Private Sector	France	3	Discontin
ETEC	Enterotoxigenic Escherichia coli (ETEC)	PPL: Critical	ACE527-102	Whole Pathogen	Live attenuated	Prophylactic	Oral	Pierrel Research USA, Inc.; Johns Hopkins Bloomberg School of Public Health	Public-Private	United States	2	Inactive
ETEC	Enterotoxigenic Escherichia coli (ETEC);	PPL: Critical	CVD 31000 (CVD 1208S-122)	Whole Pathogen	Live attenuated	Prophylactic	Oral	University of Maryland	Academia	United States	1	Active
ETEC	Enterotoxigenic Escherichia coli (ETEC)	PPL: Critical	dmLT (LTR192G/L211A)	Subunit	Recombinant	Prophylactic	Oral	National Institute of Allergy and Infectious	Government	United States	1	Active
ETEC	Enterotoxigenic Escherichia coli (ETEC)	PPL: Critical	ETVAX/dmLT; ETVAX (OEV-122); ETVAX (OEV-123); ETVAX (OEV-121); (OEV-124)	Whole Pathogen	Inactivated	Prophylactic	Oral	Scandinavian Biopharma AB	Public-Private	Sweden	2b	Active
ETEC	Enterotoxigenic Escherichia coli (ETEC)	PPL: Critical	1: CfaE +mLT (ID); CssBA+dmLT; 2: CfaE+mLT	Subunit	Recombinant	Prophylactic	Parenteral	Naval Medical Research Center; US Army Surgeon General;	Other	United States	2	Active
ETEC	Enterotoxigenic Escherichia coli (ETEC); Shigella sonnei;	PPL: Critical	Shigella-ETEC	Whole Pathogen	Live attenuated	Prophylactic	Unknown	Emergent	Private Sector	United States	1	Active
ETEC	Enterotoxigenic Escherichia coli (ETEC); Shigella sonnei;	PPL: Critical	ShigETEC	Whole Pathogen	Live attenuated	Prophylactic	Oral	EveliQure Biotechnologies GmbH	Private Sector	Austria	1	Active

ETEC	Enterotoxigenic Escherichia coli (ETEC); Salmonella enterica	PPL: Critical	TyphETEC-ZH9 typhoid–LT/ST toxoid	Subunit	Toxoid	Prophylactic	Unknown	Prokarium	Private Sector	United Kingdom	1	Inactive
ETEC	Enterotoxigenic Escherichia coli (ETEC)	PPL: Critical	VLA1701	Whole Pathogen	Inactivated	Prophylactic	Oral	Valneva Austria GmbH; Johns Hopkins University; Naval Medical Research Center	Public-Private	Austria	2	Inactive
ETEC	Enterotoxigenic Escherichia coli (ETEC)	PPL: Critical	ETEC Vaccine	Unknown	Unknown	Unknown	Unknown	Eubiologics	Private Sector	South Korea	3	Inactive
ExPEC	Extraintestinal pathogenic Escherichia	PPL: Critical	ExPEC10V (VAC52416, JNJ-69968054)	Subunit	Conjugate	Prophylactic	Parenteral	Johnson & Johnson	Private Sector	United States	1/2	Active
ExPEC	Extraintestinal pathogenic Escherichia	PPL: Critical	ExPEC4V (JNJ-63871860)	Subunit	Conjugate	Prophylactic	Parenteral	Johnson & Johnson	Private Sector	United States	2	Discontin ued
ExPEC	Extraintestinal pathogenic Escherichia	PPL: Critical	ExPEC9V	Subunit	Conjugate	Prophylactic	Parenteral	Johnson & Johnson	Private Sector	United States	3	Active
ExPEC	Extraintestinal pathogenic Escherichia coli (ExPEC)	PPL: Critical	Uro-Vaxom (OM-89)	Subunit	OMV	Prophylactic/ Therapeutic	Oral	Buckinghamshire Healthcare NHS Trust	Public-Private	United Kingdom	2	Active
ExPEC	Extraintestinal pathogenic Escherichia	PPL: Critical	UTI Vx, FimH vaccine	Subunit	Recombinant	Therapeutic	Parenteral	Sequoia Sciences	Private Sector	United States	2	Active
HiB	Haemophilus influenzae Type B	PPL: Medium	Freeze-dried Haemophilus influenzae type B combined vaccine	Unknown	Unknown	Prophylactic	Unknown	Beijing Zhifei Lvzhu Biopharmaceutic al Co., Ltd.	Private Sector	China	3	Active
HiB	Haemophilus influenzae Type B	PPL: Medium	Haemophilus Influenzae Type b (Hib) Conjugate Vaccine	Subunit	Conjugate	Prophylactic	Parenteral	Chengdu Olymvax Biopharmaceutic	Public-Private	China	3	Discontin ued
HiB	Haemophilus influenzae Type B	PPL: Medium	Hib Vaccine (Bio Farma) single shot	Unknown	Unknown	Prophylactic	Parenteral	PT Bio Farma	Private Sector	Indonesia	3	Inactive
HiB	Haemophilus influenzae Type B	PPL: Medium	Investigational Hib Vaccine (NU300)	Subunit	Conjugate	Prophylactic	Unknown	Nuron Biotech Inc.	Private Sector	United States	2	Discontin ued
HiB	Haemophilus	PPL: Medium	LBVD	Unknown	Unknown	Prophylactic	Unknown	LG Chem	Private Sector	South Korea	1	Active
HiB	Haemophilus influenzae Type B	PPL: Medium	MT-2355 (BK1310)	Subunit	Conjugate	Prophylactic	Parenteral	Mitsubishi Tanabe Pharma	Private Sector	Japan	3	Active
HiB	Haemophilus	PPL: Medium	Shan 6	Combination	NA	Prophylactic	Unknown	Sanofi Pasteur	Private Sector	France	3	Active
HiB	Haemophilus	PPL: Medium	VN-0105	Combination	NA	Prophylactic	Unknown	Daiichi Sankyo	Private Sector	Japan	3	Discontin
H. pylori	Helicobacter pylori	PPL: High	IMX101	Subunit	Recombinant	Prophylactic/ Therapeutic	Unknown	ImevaX	Private Sector	Germany	1	Inactive
TB	Mycobacterium	TB	AdHu5Ag85A	Viral vector	Non-replicating	Prophylactic	Nasal	McMaster	Public-Private	Canada	1	Active
TB	Mycobacterium tuberculosis	TB	ChadOx1.85A MVA 85A	Viral vector	Non-replicating	Prophylactic	Unknown	University of Oxford	Academia	United Kingdom	2a	Active

TB	Mycobacterium tuberculosis	TB	AEC/BC02	Subunit	Recombinant	Prophylactic	Parenteral	AnHui Zhifei Longcom Biologic Pharmacy	Private Sector	China	1	Active
TB	Mycobacterium tuberculosis	TB	GamTBVac	Subunit	Recombinant	Prophylactic	Unknown	Russian Ministry of Health	Government	Russia	3	Active
TB	Mycobacterium tuberculosis	TB	M72/AS01E (originally GSK TB vaccine 692342 (later M72))	Subunit	Recombinant	Prophylactic	Unknown	Gates MRI	Private Sector	United States	2b	Active
TB	Mycobacterium	TB	MTBVAC	Whole	Live attenuated	Prophylactic	Parenteral	Biofabri	Public-Private	Spain	3	Active
TB	Mycobacterium	TB	DAR-901 booster	Whole	Inactivated	Prophylactic	Unknown	Dartmouth	Academia	United States	2b	Active
TB	Mycobacterium	TB	MIP/Immuvac	Whole	Inactivated	Prophylactic	Unknown	ICMR	Public-Private	India	3	Active
TB	Mycobacterium tuberculosis	TB	Aeras 422	Subunit	Recombinant	Prophylactic	Parenteral	Aeras Global TB Vaccine Foundation	Other	United States	1	Discontinued
TB	Mycobacterium	TB	H4:IC31 (Aeras-404)	Subunit	Recombinant	Prophylactic	Parenteral	Aeras	Public-Private	Global	2	Discontin
TB	Mycobacterium tuberculosis	TB	MVA85A + BCG	Viral vector	Non-replicating	Prophylactic	Parenteral	University of Oxford	Academia	United Kingdom	2	Discontinued
TB	Mycobacterium tuberculosis	TB	Ag85B-ESAT-6 + IC31	Whole Pathogen	Live attenuated	Prophylactic	Unknown	Statens Serum Institut	Academia	Denmark	1	Inactive
TB	Mycobacterium tuberculosis	TB	ID93+GLA-SE	Subunit	Recombinant	Prophylactic/ Therapeutic	Parenteral	IDRI	Private Sector	United States	2a	Active
TB	Mycobacterium tuberculosis	TB	H56:IC31 (Aeras-456)	Subunit	Recombinant	Therapeutic	Parenteral	Statens Serum Institut	Public-Private	Denmark	2b	Active
TB	Mycobacterium tuberculosis	TB	RUTI	Subunit	OMV	Therapeutic	Unknown	Archivel Farma, S.L.	Private Sector	Spain	2b	Active
TB	Mycobacterium tuberculosis	TB	TB/Flu04L	Viral vector	Replicating	Therapeutic	Unknown	Research Institute for Biological Safety Problems (RIBSP)	Academia	Kazakhstan	2a	Active
TB	Mycobacterium	TB	VPM 1002 (rBCG)	Subunit	Recombinant	Therapeutic	Unknown	SIIPL	Public-Private	India	3	Active
TB	Mycobacterium	TB	Tubivac (V7)	Whole	Inactivated	Therapeutic	Oral	Immunitor	Private Sector	Canada	3	Discontin
TB	Mycobacterium	TB	GX-70	Nucleic Acid	DNA	Unknown	Unknown	Yonsei University	Academia	South Korea	1	Discontin
TB	Mycobacterium tuberculosis	TB	KCMC-001 (HVJ-E / HSP65 DNA + IL-12 DNA vaccine)	Nucleic Acid	DNA	Unknown	Unknown	National Hospital Organization Ibaraki Higashi Hospital	Academia	Japan	1	Inactive
TB	Mycobacterium tuberculosis	TB	H1-IC31 (ESAT-6, Ag85B)	Unknown	Unknown	Unknown	Unknown	Statens Serum Institut	Academia	Denmark	2	Inactive
P.	Pseudomonas	PPL: Critical	VLA43 (IC43)	Subunit	Recombinant	Prophylactic	Oral or nasal	Valneva	Private Sector	Austria	2/3	Discontin
S. Paratyphi A	Salmonella enterica serovar Paratyphi A; Salmonella enterica	PPL: High	Entervax (previously M01ZH09)	Whole Pathogen	Live attenuated	Prophylactic	Oral	Prokarium Ltd	Private Sector	United Kingdom	2b	Active

S. Paratyphi A	Salmonella enterica serovar Paratyphi A; Salmonella enterica	PPL: High	Entervax (previously M01ZH09)	Whole Pathogen	Live attenuated	Prophylactic	Oral	Prokarium Ltd	Private Sector	United Kingdom	2b	Active
S. Paratyphi A	Salmonella enterica serovar Paratyphi A	PPL: High	CVD 1902 (VASP Vaccine against Salmonella Paratyphi A)	Whole Pathogen	Live attenuated	Prophylactic	Oral	University of Oxford	Public-Private	United Kingdom	1	Active
S. Paratyphi A	Salmonella enterica serovar Paratyphi A	PPL: High	O:2,12-TT	Subunit	Conjugate	Prophylactic	Unknown	Lanzhou Institutes of Biological	Private Sector	China	3	Active
S. Typhi	Salmonella enterica serovar Typhi	PPL: High	EuTCV	Subunit	Conjugate	Prophylactic	Parenteral	Eubiologics	Private Sector	South Korea	3	Active
S. Typhi	Salmonella enterica serovar Typhi; Enterotoxigenic	PPL: High	TyphETEC-ZH9 typhoid–LT/ST toxoid	Subunit	Toxoid	Prophylactic	Unknown	Prokarium	Private Sector	United Kingdom	2	Inactive
S. Typhi	Salmonella enterica serovar Typhi	PPL: High	Typhoid Vi conjugate vaccine	Subunit	Conjugate	Prophylactic	Unknown	Sinopharm	Private Sector	China	3	Active
S. Typhi	Salmonella enterica serovar Typhi	PPL: High	Typhvax	Subunit	Protein Capsular Matrix	Prophylactic	Parenteral	Matrivax Research and Development Corporation	Private Sector	United States	1	Inactive
S. Typhi	Salmonella enterica serovar Typhi	PPL: High	Vi-DT	Subunit	Conjugate	Prophylactic	Parenteral	PT Bio Farm	Public-Private	Indonesia	3	Active
S. Typhi	serovar Typhimurium (non-typhoidal Salmonella serovar); Salmonella enterica serovar Enteritidis (non-typhoidal Salmonella serovar); Salmonella enterica	PPL: High	CVD 1000	Subunit	Conjugate	Prophylactic	Parenteral	University of Maryland	Public-Private	United States	1	Active
NTS	serovar Typhimurium (non-typhoidal Salmonella serovar); Salmonella enterica serovar Enteritidis (non-typhoidal Salmonella serovar); Salmonella enterica	PPL: High	CVD 1000	Subunit	Conjugate	Prophylactic	Parenteral	University of Maryland	Public-Private	United States	1	Active
Shigella dysenteraie	Shigella dysenteraie	PPL: Medium	GVXN SD133	Subunit	Conjugate	Prophylactic	Parenteral	LimmaTech Biologics AG	Private Sector	Switzerland	1	Discontinued
Shigella flexneri	Shigella flexneri	PPL: Medium	CVD 1208S, CVD 1208	Whole Pathogen	Live attenuated	Prophylactic	Oral	University of Maryland	Academia	United States	2	Discontinued

Shigella flexneri	Shigella flexneri; Enterotoxigenic Escherichia coli (ETEC)	PPL: Medium	CVD 31000 (CVD 1208S-122)	Whole Pathogen	Live attenuated	Prophylactic	Oral	University of Maryland	Academia	United States	1	Active
Shigella flexneri	Shigella flexneri	PPL: Medium	Flexyn2a	Subunit	Conjugate	Prophylactic	Parenteral	LimmaTech Biologics AG	Private Sector	Switzerland	2b	Inactive
Shigella	Shigella flexneri	PPL: Medium	GlycoShig3 (SF2a-TT15)	Subunit	Conjugate	Prophylactic	Parenteral	Institut Pasteur	Academia	France	2	Active
Shigella flexneri	Shigella flexneri	PPL: Medium	InvaplexAR; InvaplexAR-DETOX	Subunit	Recombinant	Prophylactic	Parenteral	PATH	Other	United States	1	Active
Shigella	Shigella flexneri	PPL: Medium	Sf2aWC + dmLT	Whole	Inactivated	Prophylactic	Oral	PATH	Other	United States	2	Discontin
Shigella flexneri	Shigella flexneri; Shigella sonnei; Enterotoxigenic	PPL: Medium	Shigella-ETEC	Whole Pathogen	Live attenuated	Prophylactic	Unknown	Emergent	Private Sector	United States	1	Active
Shigella flexneri	Shigella flexneri	PPL: Medium	Shigella4V (GSK4069327A)	Subunit	Conjugate	Prophylactic	Parenteral	LimmaTech Biologics AG	Private Sector	Switzerland	1	Active
Shigella sonnei	Shigella sonnei	PPL: Medium	1790GAHB, GSK3902986A/ GSK3536852A	Subunit	OMV	Prophylactic	Parenteral	GSK Vaccines Institute for Global Health	Private Sector	Italy	2	Inactive
Shigella sonnei	Shigella sonnei	PPL: Medium	O-SPC/rBRU	Subunit	Conjugate	Prophylactic	Unknown	Eunice Kennedy Shriver National Institute of Child Health and Human Development (NICHD)	Academia	United States	1	Inactive
Shigella sonnei	Shigella sonnei; Enterotoxigenic Escherichia coli (ETEC);	PPL: Medium	ShigETEC	Whole Pathogen	Live attenuated	Prophylactic	Oral	EveliQure Biotechnologies GmbH	Private Sector	Austria	1	Active
Shigella	Shigella sonnei	PPL: Medium	WRSS1	Whole	Live attenuated	Prophylactic	Oral	PATH	Other	United States	2	Inactive
Shigella sonnei	Shigella sonnei	PPL: Medium	WRSS2/WRSS3 (VirG series)	Whole Pathogen	Live attenuated	Prophylactic	Oral	National Institute of Allergy and Infectious	Other	United States	2	Active
S. aureus	Staphylococcus aureus	PPL: High	rTSST-1 Variant Vaccine (ORG28077)	Subunit	Recombinant	Prophylactic	Parenteral	Biomedizinische Forschungs gmbH	Public-Private	Austria; United States	2	Active
S. aureus	Staphylococcus aureus	PPL: High	Recombinant Staphyloccus aureus Vaccine	Subunit	Recombinant	Unknown	Unknown	Chengdu Olymvax Biopharmaceutic	Private Sector	China	1	Inactive
S. aureus	Staphylococcus aureus	PPL: High	GSK3878858A	Subunit	Recombinant	Prophylactic	Parenteral	GlaxoSmithKline, plc	Private Sector	United Kingdom	1/2	Active
S. aureus	Staphylococcus aureus	PPL: High	rAT and rLukS-PV	Subunit	Toxoid	Prophylactic	Parenteral	GlaxoSmithKline, plc	Private Sector	United States	1/2	Discontinued
S. aureus	Staphylococcus aureus	PPL: High	STEBVax (IBT-V01)	Subunit	Recombinant	Prophylactic	Parenteral	Integrated BioTherapeutics Inc.	Private Sector	United States	1	Discontinued

S. aureus	Staphylococcus aureus	PPL: High	NDV-3A (formerly NDV3)	Subunit	Recombinant	Prophylactic	Parenteral	NovaDigm Therapeutics Inc.	Private Sector	United States	2	Discontinued
S. aureus	Staphylococcus aureus	PPL: High	SA4Ag (Staphylococcus aureus 4-antigen vaccine) PF-06290510/SA4Ag	Subunit	Conjugate	Prophylactic	Parenteral	Pfizer	Private Sector	United States	2b	Discontinued
S. aureus	Staphylococcus aureus	PPL: High	V710 IsdB	Subunit	Recombinant	Prophylactic	Parenteral	Merck Sharp & Dohme Corp.	Private Sector	United States	3	Discontinued
S. aureus	Staphylococcus aureus	PPL: High	4C-Staph	Subunit	Toxoid	Prophylactic	Parenteral	GlaxoSmithKline, plc	Private Sector	United Kingdom	1	Discontinued
S. aureus	Staphylococcus aureus; Streptococcus	PPL: High	AV0328	Subunit	Conjugate	Prophylactic	Parenteral	Alopexx Vaccine, LLC	Private Sector	United States	1/2	Inactive
S. aureus	Staphylococcus aureus	PPL: High	SA75	Whole Pathogen	Inactivated	Prophylactic	Unknown	Vaccine Research International	Academia	United Kingdom	1	Inactive
S.	Streptococcus	PPL: Medium	PF-06842433	Unknown	Unknown	Prophylactic	Unknown	Pfizer	Private Sector	United States	2	Active
S. pneumoniae	Streptococcus pneumoniae	PPL: Medium	15 Valent Pneumococcal conjugate Vaccine	Subunit	Conjugate	Prophylactic	Parenteral	Tergene Biotech; Aurobindo Pharma	Private Sector	India	2	Active
S. pneumoniae	Streptococcus pneumoniae	PPL: Medium	15-Valent Pneumococcal Conjugate Vaccine	Subunit	Conjugate	Prophylactic	Parenteral	Beijing Zhifei Lvzhu Biopharmaceutical Co., Ltd	Private Sector	China	3	Active
S. pneumoniae	Streptococcus pneumoniae	PPL: Medium	23-valent pneumococcal polysaccharide vaccine ppsv23	Subunit	Unknown	Prophylactic	Parenteral	Beijing Zhifei Lvzhu Biopharmaceutical Co., Ltd	Private Sector	China	3	Active
S. pneumoniae	Streptococcus pneumoniae	PPL: Medium	ASP3772	Subunit	Multiple Antigen Presenting	Prophylactic	Parenteral	Astellas Pharma Inc	Private Sector	Japan	2	Active
S. pneumoniae	Streptococcus pneumoniae;	PPL: Medium	AV0328	Subunit	Conjugate	Prophylactic	Parenteral	Alopexx Vaccine, LLC	Private Sector	United States	1/2	Inactive
S. pneumoniae	Streptococcus pneumoniae	PPL: Medium	Bioconjugate pneumococcal vaccine	Subunit	Conjugate	Prophylactic	Unknown	LimmaTech Biologics AG	Private Sector	Switzerland	1	Inactive
S.	Streptococcus	PPL: Medium	Nucovac	Subunit	Conjugate	Prophylactic	Parenteral	Panacea Biotec	Private Sector	India	2	Active
S. pneumoniae	Streptococcus pneumoniae	PPL: Medium	PCV13a	Subunit	Conjugate	Prophylactic	Parenteral	Beijing Chaoyang District Centre for Disease Control and	Government	China	1	Inactive
S. pneumoniae	Streptococcus pneumoniae	PPL: Medium	PCV7 (VCN7-T)	Subunit	Conjugate	Prophylactic	Unknown	Biomolecular Chemistry Center (CQB)	Academia	Cuba	2/3	Inactive
S. pneumoniae	Streptococcus pneumoniae	PPL: Medium	Pneumococcal 13-valent Conjugate Vaccine (PCV)	Subunit	Conjugate	Prophylactic	Unknown	Tianjin CanSino Biotechnology	Private Sector	China	1	Active

S. pneumoniae	Streptococcus pneumoniae	PPL: Medium	Pneumococcal conjugate vaccine 14-valent (adsorbed)	Subunit	Conjugate	Prophylactic	Parenteral	Biological E	Private Sector	India	3	Active
S. pneumoniae	Streptococcus pneumoniae	PPL: Medium	Pneumococcal Protein Vaccine (PPrV)	Subunit	Recombinant	Prophylactic	Parenteral	Sanofi Pasteur	Private Sector	France	2	Active
S. pneumoniae	Streptococcus pneumoniae	PPL: Medium	Pneumococcal vaccine conjugate 13-valent	Subunit	Conjugate	Prophylactic	Parenteral	Beijing Minhai Biotechnology	Private Sector	China	3	Active
S. pneumoniae	Streptococcus pneumoniae	PPL: Medium	PneumoNexgen Vaccine Multivalent Pneumococcal Conjugate Vaccine (PCV20)	Subunit	Conjugate	Prophylactic	Parenteral	Pfizer	Private Sector	United States	2	Discontinued
S. pneumoniae	Streptococcus pneumoniae	PPL: Medium	PnuBiovax (PBV)	Subunit	Toxoid	Prophylactic	Parenteral	ImmunoBiology Limited	Private Sector	United Kingdom	1	Inactive
S. pneumoniae	Streptococcus pneumoniae	PPL: Medium	Polyvalent Pneumococcal Conjugate Vaccine (pPCV) V116	Subunit	Conjugate	Prophylactic	Unknown	Merck Sharp & Dohme Corp.	Private Sector	United States	2	Active
S. pneumoniae	Streptococcus pneumoniae	PPL: Medium	Protein based pneumococcal vaccine (PBPV)	Subunit	Conjugate	Prophylactic	Parenteral	CanSino Biologics Inc..	Public-Private	China	1	Active
S. pneumoniae	Streptococcus pneumoniae	PPL: Medium	RASV (recombinant avirulent salmonella typhi)	Subunit	Recombinant	Prophylactic	Oral	Arizona State University	Academia	United States	1	Inactive
S.	Streptococcus	PPL: Medium	SP0202, Skypac	Subunit	Conjugate	Prophylactic	Parenteral	Sanofi Pasteur	Private Sector	France	2	Active
S. pneumoniae	Streptococcus pneumoniae	PPL: Medium	Streptococcus Pneumoniae Whole Cell Vaccine (SPWCV) + Alum	Whole Pathogen	Inactivated	Prophylactic	Parenteral	PATH	Public-Private	United States	1/2	Inactive
S. pneumoniae	Streptococcus pneumoniae	PPL: Medium	13-Valent Pneumococcal conjugate Vaccine	Subunit	Conjugate	Unknown	Unknown	Sinopharm	Private Sector	China	1	Active
S. pneumoniae	Streptococcus pneumoniae	PPL: Medium	dPly (pneumolysoid)/PhtDw/PHiD-CV (GSK2189242A)	Subunit	Conjugate	Unknown	Unknown	GlaxoSmithKline, plc	Private Sector	United Kingdom	2	Discontinued
S.	Streptococcus	PPL: Medium	euPCV	Subunit	Conjugate	Unknown	Unknown	EuBiologics C. Ltd	Private Sector	South Korea	1	Active
S. pneumoniae	Streptococcus pneumoniae	PPL: Medium	GEN-004, SP0148, 1912, 2108	Subunit	Recombinant	Unknown	Unknown	Genocea Biosciences, Inc.	Private Sector	United States	3	Discontinued
S. pneumoniae	Streptococcus pneumoniae	PPL: Medium	IC47	Subunit	Recombinant	Unknown	Unknown	Valneva Austria GmbH	Private Sector	Austria	1	Inactive
S.	Streptococcus	PPL: Medium	PCV (LBVE)	Subunit	Conjugate	Unknown	Unknown	LG Chem	Private Sector	South Korea	2	Active
S. pneumoniae	Streptococcus pneumoniae	PPL: Medium	Pneumococcal Vaccine Program	Unknown	Unknown	Unknown	Unknown	Instituto Butantan	Academia	Brazil	1	Inactive
K. pneumoniae	Klebsiella pneumoniae	PPL: Critical	KlebV4	Subunit	Conjugate	Prophylactic	Unknown	GlaxoSmithKline, plc	Private Sector	United Kingdom	1/2	Active
N.	Neisseria gonorrhoeae	PPL: High	4CMenB (Bexsero)	Subunit	Conjugate	Prophylactic	Parenteral	Kirby Institute	Academia	Australia	3	Active

Shigella flexneri	Shigella flexneri; Shigella sonnei	PPL: Medium	Multivalent Shigella GMMA	Subunit	GMMA	Prophylactic	Parenteral	GSK Vaccines Institute for Global Health (GVGH)	Private Sector	Italy	1	Active
Shigella sonnei	Shigella flexneri; Shigella sonnei	PPL: Medium	Multivalent Shigella GMMA	Subunit	GMMA	Prophylactic	Parenteral	GSK Vaccines Institute for Global Health (GVGH)	Private Sector	Italy	1	Active

Appendix

Preclinical data

Pathogen	All Pathogens	multi-pathogen	Pathogen Type	Candidate Vaccine	Developer Name	Developer Type	Developer Country	Activity Status
S. aureus	Staphylococcus aureus; Enterotoxigenic Escherichia coli (ETEC); Extraintestinal pathogenic Escherichia coli (ExPEC)	y	PPL: High	Paragon Novel Vaccine (PNV)	Immunethep	Private Sector	Portugal	Active
S. aureus	Staphylococcus aureus	n	PPL: High	IBT-V02	Integrated BioTherapeutics, US	Private Sector	United States	Active
S. aureus	Staphylococcus aureus	n	PPL: High	Staphyloccus aureus Vaccine	Syntiron	Private Sector	United States	Active
S. aureus	Staphylococcus aureus	n	PPL: High	MRSA Vaccine Project	VLP Biotech	Private Sector	United States	Unknown
S. aureus	Staphylococcus aureus	n	PPL: High	Staphylococcus aureus vaccine	Abcombi Biosciences	Private Sector	United States	Active
S. aureus	Staphylococcus aureus	n	PPL: High	Staphylococcus Aureus Vaccine Program	Astrogenetix	Private Sector	United States	Active
S. aureus	Staphylococcus aureus	n	PPL: High	Staphylococcus Aureus Vaccine Project	Affinivax	Private Sector	United States	Active
S. aureus	Staphylococcus aureus	n	PPL: High	SAG (Staphylococcus aureus Ghosts)	Pan Chai University	Academia	China	Unknown
S. aureus	Staphylococcus aureus	n	PPL: High	EVX-B1	Evaxion	Private Sector	United States	Active
S. aureus	Staphylococcus aureus	n	PPL: High	ABN-701	Abion	Private Sector	South Korea	Active
S. aureus	Staphylococcus aureus	n	PPL: High	CTI-005	Cellics Therapeutics	Private Sector	United States	Active
S. aureus	Staphylococcus aureus	n	PPL: High	CTI-036	Cellics Therapeutics	Private Sector	United States	Active
S. aureus	Staphylococcus aureus	n	PPL: High	S. aureus vaccine Cologne	University of Cologne	Academia	Germany	Active
S. aureus	Staphylococcus aureus	n	PPL: High	S. aureus vaccine Janssen	Janssen Vaccines and Prevention	Private Sector	Netherlands	Active
C. difficile	Clostridiodes Difficile	n	C. difficile	Clostridium difficile vaccine Astellas Pharma	Affinivax	Private Sector	United States	Active
C. difficile	Clostridiodes Difficile	n	C. difficile	Clostridium difficile vaccine ImmBio	ImmBio	Private Sector	United Kingdom	Active

C. difficile	Clostridiodes Difficile	n	C. difficile	MVX02 Clostridium difficile vaccine	Matrivax Corporation	Private Sector	United States	Active
C. difficile	Clostridiodes Difficile	n	C. difficile	Clostridium difficile vaccine Versatope	Versatope-Therapeutics	Private Sector	United States	Active
C. difficile	Clostridiodes Difficile	n	C. difficile	SporeVax Clostridium difficile oral vaccine	SporeGen	Private Sector	United Kingdom	Unknown
C. jejuni	Campylobacter jejuni; Enterotoxigenic Escherichia coli (ETEC)	y	PPL: High	Campylobacter/ ETEC Vaccines Immuron	Immuron	Public-Private	Australia	Active
C. jejuni	Campylobacter jejuni; Enterotoxigenic Escherichia coli (ETEC); Shigella flexneri	y	PPL: High	Glycoconjugate vaccine	Naval Medical Research Center	Government	United States	Active
C. jejuni	Campylobacter jejuni	n	PPL: High	PEB1 DNA prime/ protein boost	Shandong Medical College	Academia	China	Unknown
C. jejuni	Campylobacter jejuni	n	PPL: High	CJCV2	U.S. Naval Medical Research Unit	Government	United States	Active
ETEC	Staphylococcus aureus; Enterotoxigenic Escherichia coli (ETEC); Extraintestinal pathogenic Escherichia coli (ExPEC)	y	PPL: Critical	Paragon Novel Vaccine (PNV)	Immunethep	Private Sector	Portugal	Active
ETEC	Enterotoxigenic Escherichia coli (ETEC)	n	PPL: Critical	ETEC vaccine Hilleman	Hilleman Laboratories	Private Sector	India	Unknown
ETEC	Enterotoxigenic Escherichia coli (ETEC)	n	PPL: Critical	MecVax (Multi-Epitope Antigen (MEFA) vaccine)	University of Illinois	Academia	United States	Active
ETEC	Enterotoxigenic Escherichia coli (ETEC)	n	PPL: Critical	LT/ST toxoids	University of Maryland	Academia	United States	Unknown
ETEC	Enterotoxigenic Escherichia coli (ETEC)	n	PPL: Critical	Fimbrial tip adhesins (FTA)	PATH	Other	United States	Unknown
ETEC	Enterotoxigenic Escherichia coli (ETEC)	n	PPL: Critical	ETEC vaccine Bergen	University of Bergen	Academia	Norway	Active
ETEC	Enterotoxigenic Escherichia coli (ETEC)	n	PPL: Critical	ETEC vaccine Washingto n	Washington University School of Medicine	Academia	United States	Active

ETEC	Campylobacter jejuni; Enterotoxigenic Escherichia coli (ETEC)	y	PPL: Critical	Campylobacter/ ETEC Vaccines Immuron	Immuron	Public-Private	Australia	Active
ETEC	Campylobacter jejuni; Enterotoxigenic Escherichia coli (ETEC); Shigella flexneri	y	PPL: Critical	Glycoconjugate vaccine	Naval Medical Research Center	Government	United States	Active
ETEC	Enterotoxigenic Escherichia coli (ETEC); Extraintestinal pathogenic Escherichia coli (ExPEC)	y	PPL: Critical	Glycoprotein based candidate	GlyProVac	Private Sector	Denmark	Active
ExPEC	Staphylococcus aureus; Enterotoxigenic Escherichia coli (ETEC); Extraintestinal pathogenic Escherichia coli (ExPEC)	y	PPL: Critical	Paragon Novel Vaccine (PNV)	Immunethep	Private Sector	Portugal	Active
ExPEC	Extraintestinal pathogenic Escherichia coli (ExPEC)	n	PPL: Critical	ExPEC vaccine	Pfizer	Private Sector	United States	Active
ExPEC	Enterotoxigenic Escherichia coli (ETEC); Extraintestinal pathogenic Escherichia coli (ExPEC)	y	PPL: Critical	Glycoprotein based candidate	GlyProVac	Private Sector	Denmark	Active
ExPEC	Extraintestinal pathogenic Escherichia coli (ExPEC); Klebsiella pneumoniae	y	PPL: Critical	E. coli and Klebsiella vaccine	Syntiron	Private Sector	United States	Active
Shigella spp	Campylobacter jejuni; Enterotoxigenic Escherichia coli (ETEC); Shigella flexneri	y	PPL: Medium	Glycoconjugate vaccine	Naval Medical Research Center	Government	United States	Active
Shigella spp	Shigella sonnei; Shigella flexneri	y	PPL: Medium	DB Fusion. Serotype-independent Shigella vaccine candidate	Oklahoma State University	Other	United States	Active
Shigella spp	Shigella sonnei; Shigella flexneri	y	PPL: Medium	ShigOraVax	ShigOraVax Consortium	Public-Private	Global	Active

Shigella spp	Shigella flexneri	n	PPL: Medium	34 kDa OMP	National Institute of Cholera and Enteric Diseases	Academia	India	Active
Shigella spp	Shigella sonnei; Shigella dysenteraieShigella flexneri	y	PPL: Medium	Truncated mutant	International Vaccine Institute	Academia	South Korea	Active
Shigella spp	Shigella flexneri	n	PPL: Medium	OMV Sfl2a	University of Navarra	Academia	Spain	Active
Shigella spp	Shigella sonnei; Shigella flexneri; Shigella dysenteraie	y	PPL: Medium	PSSP-1	International Vaccine Institute	Academia	South Korea	Active
Shigella spp	Shigella sonnei; Shigella flexneri; Shigella dysenteraie	y	PPL: Medium	HKMS	National Institute of Cholera and Enteric Diseases	Academia	India	Active
Shigella spp	Salmonella enterica serovar Typhi; Shigella sonnei; Shigella flexneri	y	PPL: Medium	Recombinant Ty21a typhoid vaccine expressing Shigella LPS	Protein Potential LLC	Private Sector	United States	Active
Shigella spp	Shigella sonnei; Shigella flexneri	y	PPL: Medium	Shigella flexneri 2a and Sonnei conjugate	Zhifei	Private Sector	China	Active
S. Typhi	Salmonella enterica serovar Typhimurium (non-typhoidal Salmonella serovar); Salmonella enterica serovar Typhi	y	PPL: High	iNTS-TCV vaccine	GSK Vaccines Institute for Global Health Srl (GVGH)	Private Sector	Italy	Active
S. Typhi	Salmonella enterica serovar Typhimurium (non-typhoidal Salmonella serovar); Salmonella enterica serovar Enteritidis (non-typhoidal Salmonella serovar); Salmonella enterica serovar Typhi	y	PPL: High	Trivalent conjugate vaccine	SK Bioscience	Private Sector	South Korea	Active

S. Typhi	Salmonella enterica serovar Typhimurium (non-typhoidal Salmonella serovar); Salmonella enterica serovar Enteritidis (non-typhoidal Salmonella serovar); Salmonella enterica serovar Typhi	y	PPL: High	iNTS-GMMA	GSK Vaccines Institute for Global Health (GVGH)	Private Sector	Italy	Active
S. Typhi	Salmonella enterica serovar Typhi	n	PPL: High	Vi-PsaA-PdT	Harvard Medical School	Academia	United States	Active
S. Typhi	Salmonella enterica serovar Paratyphi A; Salmonella enterica serovar Typhi	y	PPL: High	O:2,12-DT + Vi-DT	International Vaccine Institute	Academia	South Korea	Active
S. Typhi	Salmonella enterica serovar Paratyphi A; Salmonella enterica serovar Typhi	y	PPL: High	O:2,12-CRM197 + Vi-CRM197	GSK Vaccines Institute for Global Health	Private Sector	Italy	Active
S. Typhi	Salmonella enterica serovar Paratyphi A; Salmonella enterica serovar Typhi	y	PPL: High	Salmonella Typhi and Paratyphi vaccine	University of Oxford	Academia	United Kingdom	Active
S. Typhi	Salmonella enterica serovar Typhi; Shigella sonnei; Shigella flexneri	y	PPL: High	Recombinant Ty21a typhoid vaccine expressing Shigella LPS	Protein Potential LLC	Private Sector	United States	Active
S. Paratyphi A	Salmonella enterica serovar Paratyphi A; Salmonella enterica serovar Typhi	y	PPL: High	O:2,12-DT + Vi-DT	International Vaccine Institute	Private Sector	South Korea	Active
S. Paratyphi A	Salmonella enterica serovar Paratyphi A; Salmonella enterica serovar Typhi	y	PPL: High	O:2,12-CRM197 + Vi-CRM197	GSK Vaccines Institute for Global Health	Private Sector	Italy	Active
S. Paratyphi A	Salmonella enterica serovar Paratyphi A; Salmonella enterica serovar Typhi	y	PPL: High	Salmonella Typhi and Paratyphi vaccine	University of Oxford	Academia	United Kingdom	Active

NTS	Salmonella enterica serovar Typhimurium (non-typhoidal Salmonella serovar); Salmonella enterica serovar Typhi	y	PPL: High	iNTS-TCV vaccine	GSK Vaccines Institute for Global Health Srl (GVGH)	Private Sector	Italy	Active
NTS	Salmonella enterica serovar Typhimurium (non-typhoidal Salmonella serovar); Salmonella enterica serovar Enteritidis (non-typhoidal Salmonella serovar); Salmonella enterica serovar Typhi	y	PPL: High	Trivalent conjugate vaccine	SK Bioscience	Private Sector	South Korea	Active
NTS	Salmonella enterica serovar Typhimurium (non-typhoidal Salmonella serovar); Salmonella enterica serovar Enteritidis (non-typhoidal Salmonella serovar); Salmonella enterica serovar Typhi	y	PPL: High	iNTS-GMMA	GSK Vaccines Institute for Global Health (GVGH)	Private Sector	Italy	Active
NTS	Salmonella enterica serovar Typhimurium (non-typhoidal Salmonella serovar); Salmonella enterica serovar Enteritidis (non-typhoidal Salmonella serovar)	y	PPL: High	CVD 1944 (derived from S. Enteritidis) and CVD 1931 (derived form S. Typhimurium)	University of Maryland	Academia	United States	Active
NTS	Salmonella enterica serovar Paratyphi A	n	PPL: High	S. Paratyphi A vaccine candidate	KJ Biosciences	Private Sector	United States	Active
NTS	Salmonella enterica serovar Typhimurium (non-typhoidal Salmonella serovar)	n	PPL: High	OmpD	University of Birmingham	Academia	United Kingdom	Active

HiB	Haemophilus influenzae Type B	n	PPL: Medium	DTcP-Hib Combo Vaccine and MCV2-Hib Combination Vaccine	Tianjin Cansino Biotechnology	Private Sector	China	Active
HiB	Haemophilus influenzae Type B	n	PPL: Medium	Haemopilus influenzae Vaccine Wellstat	Wellstat Therapeutics	Private Sector	United States	Active
HiB	Haemophilus influenzae Type B	n	PPL: Medium	Haemopilus influenzae Vaccine HanaVax	HanaVax	Private Sector	Japan	Active
H. pylori	Helicobacter pylori	n	PPL: High	Probiotic vaccine delivery	China Pharmaceutical University	Academia	China	Unknown
H. pylori	Helicobacter pylori	n	PPL: High	H. pylori vaccine EpiVax	EpiVax	Private Sector	United States	Inactive
H. pylori	Helicobacter pylori	n	PPL: High	H. pylori vaccine ImmBio	ImmBio	Private Sector	United Kingdom	Inactive
H. pylori	Helicobacter pylori	n	PPL: High	Inactivated whole cell vaccine	University of Gothenburg	Academia	Sweden	Inactive
H. pylori	Helicobacter pylori	n	PPL: High	H. pylori surfome antigens	Technical University Munich	Academia	Germany	Active
H. pylori	Helicobacter pylori	n	PPL: High	Gastric Cancer Vaccine	MCRI (Murdoch Children's Research Institute)	Academia	Australia	Active
P. aeruginosa	Klebsiella pneumoniae; Pseudomonas aeruginosa	y	PPL: Critical	ASP1004	Affinivax	Private Sector	United States	Active
P. aeruginosa	Pseudomonas aeruginosa	n	PPL: Critical	INI-2002	Inimmune	Private Sector	United States	Active
P. aeruginosa	Pseudomonas aeruginosa	n	PPL: Critical	OprF and OprL	Unknown	Unknown	China	Active
P. aeruginosa	Acinetobacter baumannii; Pseudomonas aeruginosa; Klebsiella pneumonia	y	PPL: Critical	KapaVax (VXD-005)	Vaxdyn	Private Sector	Spain	Active
S. pneumoniae	Streptococcus pneumoniae	n	PPL: Medium	Pneumococcal Vaccine Abera	Abera Bioscient	Private Sector	Sweden	Active

S. pneumoniae	Streptococcus pneumoniae	n		PPL: Medium	Streptococcus Pneumoniae Research Project (next generation) Affinivax	Affinivax	Private Sector	United States	Active
S. pneumoniae	Streptococcus pneumoniae	n		PPL: Medium	Streptococcus Pneumoniae Research Project (next generation) Astrogenetix	Astrogenetix	Private Sector	United States	Unknown
S. pneumoniae	Streptococcus pneumoniae	n		PPL: Medium	13-Valent Pneumococcal conjugate Vaccine	Zhifei	Private Sector	China	Active
S. pneumoniae	Streptococcus pneumoniae	n		PPL: Medium	Pneumococcal Vaccine Eurocine	Eurocine Vaccines	Private Sector	Sweden	Active
S. pneumoniae	Streptococcus pneumoniae	n		PPL: Medium	Pneumococcal Vaccine Gamma	Gamma Vaccines	Private Sector	Australia	Active
S. pneumoniae	Streptococcus pneumoniae	n		PPL: Medium	Pneumococcal 13-valent Conjugate Vaccine (CRM197,TT, PCV 13i)	Sinovac Biotech	Private Sector	China	Unknown
S. pneumoniae	Streptococcus pneumoniae	n		PPL: Medium	VAX-24	Vaxcyte	Private Sector	United States	Active
S. pneumoniae	Streptococcus pneumoniae	n		PPL: Medium	VAX-XP	Vaxcyte	Private Sector	United States	Active
S. pneumoniae	Streptococcus pneumoniae	n		PPL: Medium	Pneumococcal Vaccine Virometix	Virometix	Private Sector	Switzerland	Inactive
S. pneumoniae	Streptococcus pneumoniae	n		PPL: Medium	Pneumococcal Vaccine Wellstat	Wellstat Therapeutics	Private Sector	United States	Active
S. pneumoniae	Streptococcus pneumoniae	n		PPL: Medium	Pneumococcal Vaccine Abcombi	Abcombi Biosciences	Private Sector	United States	Active
S. pneumoniae	Streptococcus pneumoniae	n		PPL: Medium	Pneumococcal Vaccine Synovac	Synovac	Private Sector	China	Active
S. pneumoniae	Streptococcus pneumoniae	n		PPL: Medium	Pneumococcal Vaccine HanaVax	HanaVax	Private Sector	Japan	Active
S. pneumoniae	Streptococcus pneumoniae	n		PPL: Medium	MVX01	Matrivax Corporation	Private Sector	United States	Active
S. pneumoniae	Streptococcus pneumoniae	n		PPL: Medium	PnuVax	PnuVax	Private Sector	Canada	Unknown

S. pneumoniae	Streptococcus pneumoniae	n	PPL: Medium	CMTX-301	Clarametyx Biosciences	Private Sector	United Kingdom	Active
TB	Mycobacterium tuberculosis	n	TB	Tuberculosis Vaccine EpiVax	EpiVax	Private Sector	United States	Active
TB	Mycobacterium tuberculosis	n	TB	GI-19000 Tuberculosis Vaccine	GlobeImmune	Private Sector	United States	Active
TB	Mycobacterium tuberculosis	n	TB	T-Bio vax	ImmBio	Private Sector	United Kingdom	Active
TB	Mycobacterium tuberculosis	n	TB	Lipovax-Fg115-TB	Lipotek	Private Sector	Australia	Active
TB	Mycobacterium tuberculosis	n	TB	Lipovax-FliC-TB	Lipotek	Private Sector	Australia	Active
TB	Mycobacterium tuberculosis	n	TB	Tuberculosis Research Programme	Longhorn Vaccines and Diagnostics	Private Sector	United States	Active
TB	Mycobacterium tuberculosis	n	TB	Therapeutic MDR Tuberculosis Programme	Theravectys	Private Sector	France	Active
TB	Mycobacterium tuberculosis	n	TB	TVI-Tuberculosis-1	TVAX Biomedical	Private Sector	United States	Active
TB	Mycobacterium tuberculosis	n	TB	MTbuVax	Vaxil BioTherapeutics	Private Sector	Israel	Active
TB	Mycobacterium tuberculosis	n	TB	Tuberculosis Vaccine Greffex	Greffex	Private Sector	United States	Active
TB	Mycobacterium tuberculosis	n	TB	Tuberculosis Vaccine Janssen	Janssen	Private Sector	Belgium	Active
TB	Mycobacterium tuberculosis	n	TB	Tuberculosis Vaccine HanaVax	HanaVax	Private Sector	Japan	Active
TB	Mycobacterium tuberculosis	n	TB	H107	Staten Serum Institute	Public-Private	Denmark	Active
TB	Mycobacterium tuberculosis	n	TB	CysVac2/Ad	University of Sydney	Public-Private	Australia	Active
TB	Mycobacterium tuberculosis	n	TB	MVA Multivac.	Transgene	Public-Private	France	Active
TB	Mycobacterium tuberculosis	n	TB	BCG-ZMP1	University of Zurich	Public-Private	Switzerland	Active
TB	Mycobacterium tuberculosis	n	TB	BCG, ChadOx/MVA PPE15-85A	University of Oxford	Public-Private	United Kingdom	Active
TB	Mycobacterium tuberculosis	n	TB	Tuberculosis Vaccine BioNTech	BioNTech	Private Sector	Germany	Active
TB	Mycobacterium tuberculosis	n	TB	PDS0201	PDS Biotechnology	Private Sector	United States	Active

TB	Mycobacterium tuberculosis	n	TB	CMV-TB	Vir Biotechnology	Private Sector	United States	Active
A. baumannii	Acinetobacter baumannii	n	PPL: Critical	A. baumannii and S. agalactiae conjugate vaccine	Bio-Manguinhos	Private Sector	Brazil	Active
A. baumannii	Acinetobacter baumannii	n	PPL: Critical	Acineto Vax (VXD-001)	Vaxdyn	Private Sector	Spain	Active
A. baumannii	Acinetobacter baumannii	n	PPL: Critical	A. baumannii vaccine Oswaldo	Oswaldo Cruz Foundation	Academia	Brazil	Active
A. baumannii	Acinetobacter baumannii	n	PPL: Critical	A. baumannii vaccine Birmingham	University of Birmingham	Academia	United Kingdom	Active
A. baumannii	Acinetobacter baumannii; Pseudomonas aeruginosa; Klebsiella pneumoniae	y	PPL: Critical	KapaVax (VXD-005)	Vaxdyn	Private Sector	Spain	Active
K. pneumoniae	Klebsiella pneumoniae	n	PPL: Critical	VXN-319	Idorsia Pharmaceuticals (previously Vaxxilon AG)	Private Sector	Switzerland	Active
K. pneumoniae	Klebsiella pneumoniae; Pseudomonas aeruginosa	y	PPL: Critical	ASP1004	Affinivax	Private Sector	United States	Active
K. pneumoniae	Extraintestinal pathogenic Escherichia coli (ExPEC); Klebsiella pneumoniae	y	PPL: Critical	E. coli and Klebsiella vaccine	Syntiron	Private Sector	United States	Active
K. pneumoniae	Acinetobacter baumannii; Pseudomonas aeruginosa; Klebsiella pneumoniae	y	PPL: Critical	KapaVax (VXD-005)	Vaxdyn	Private Sector	Spain	Active
N. gonorrhoeae	Neisseria gonorrhoeae	n	PPL: High	NGoXIM	Therapyx	Private Sector	Netherlands	Active
N. gonorrhoeae	Neisseria gonorrhoeae	n	PPL: High	dmGC_0817560 NOMV	Oxford Vaccine Group	Academia	United Kingdom	Active
K. pneumoniae	Klebsiella pneumoniae	n	PPL: Critical	K pneumoniae vaccine	Tulane University	Academia	United States	Active
